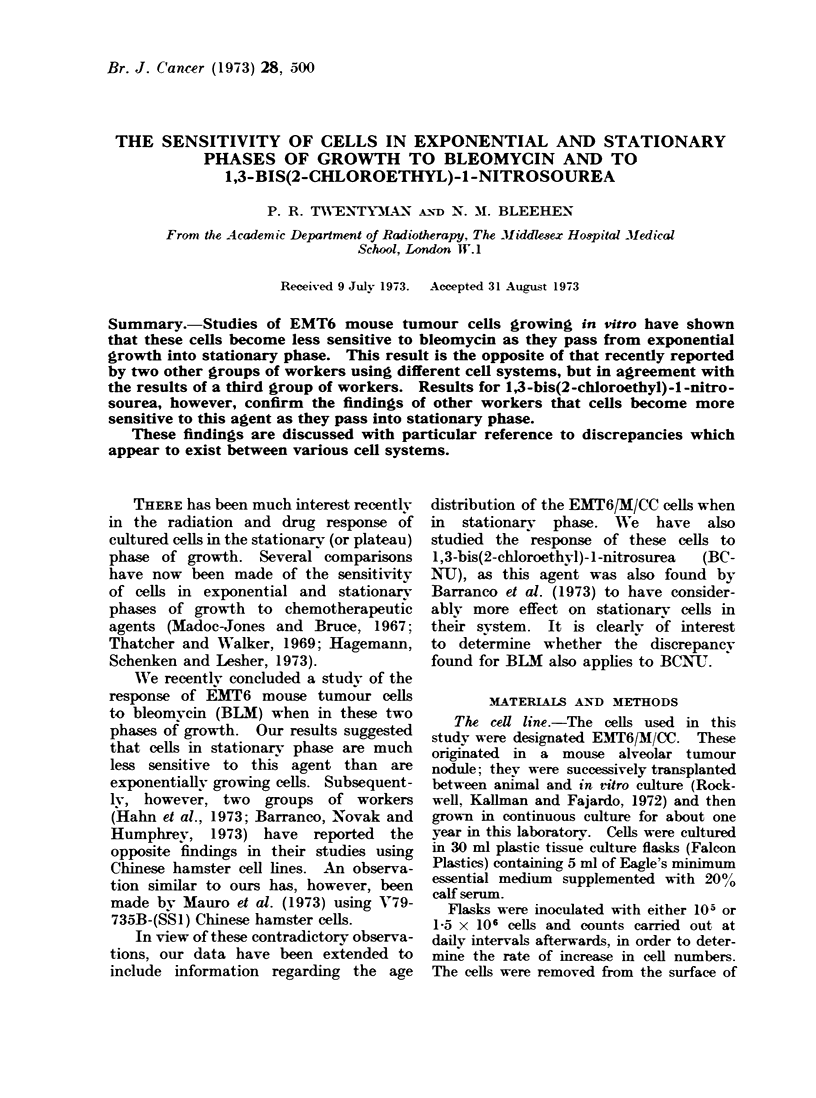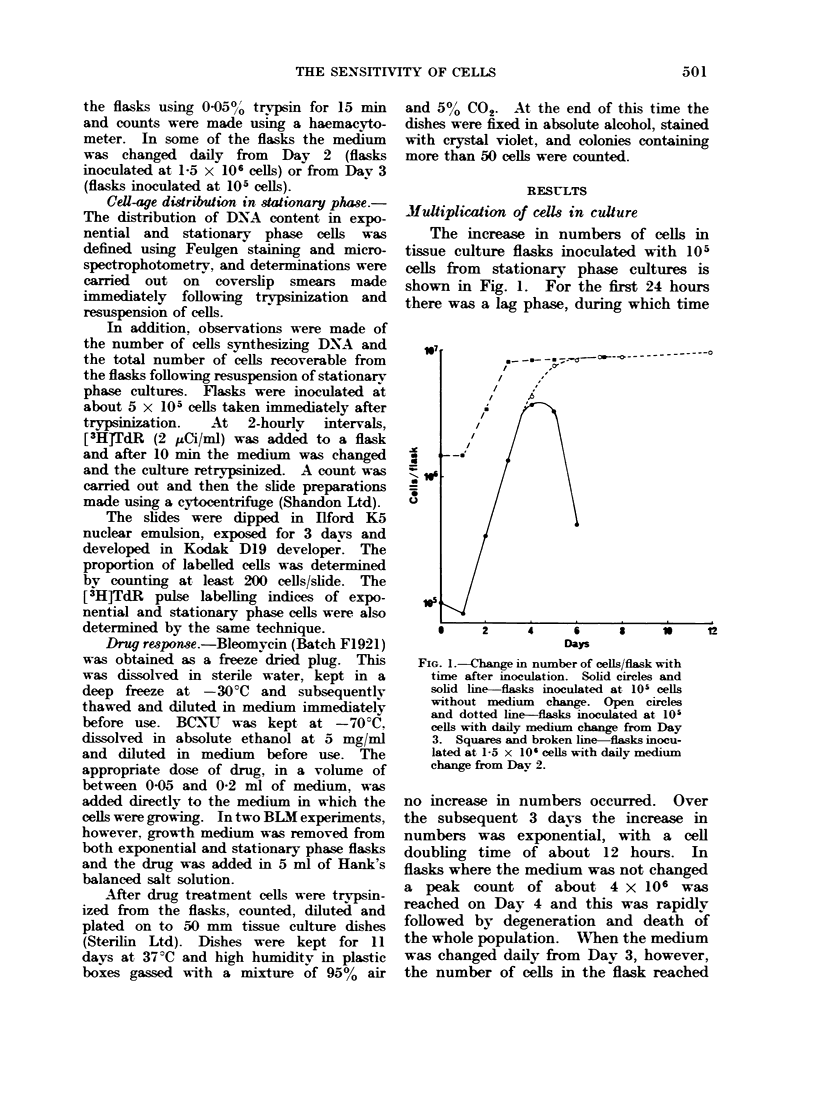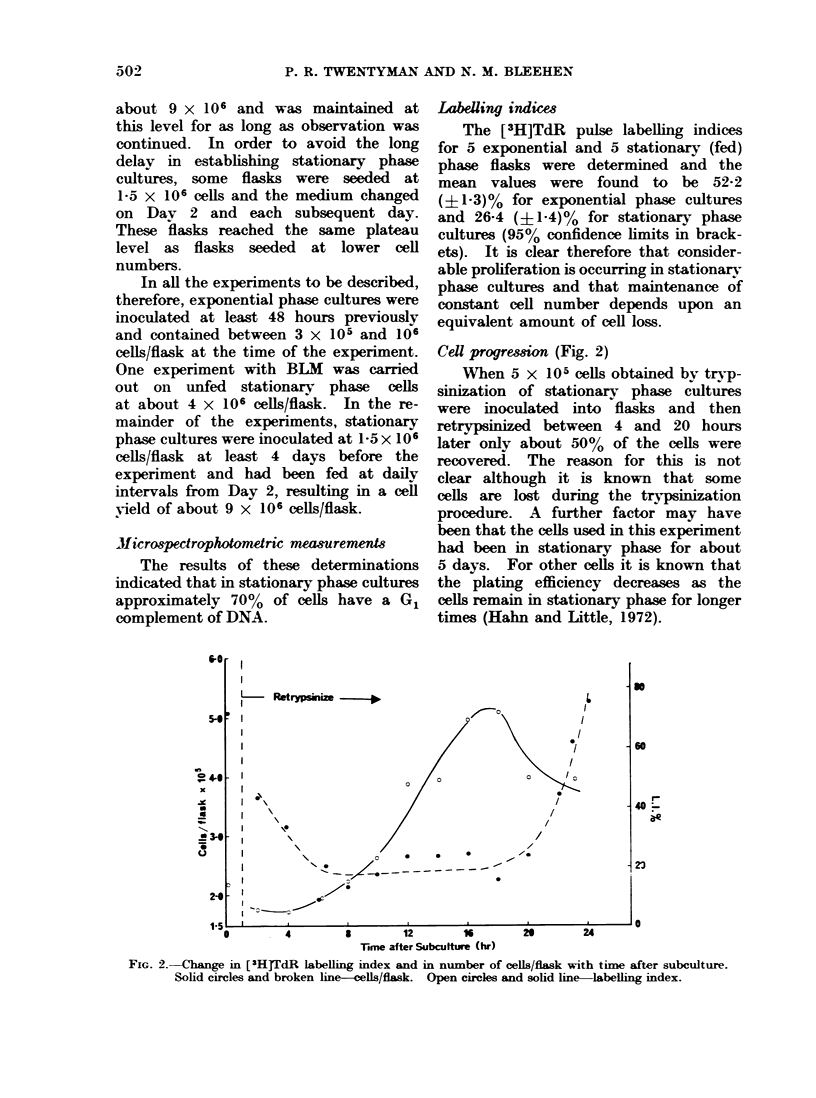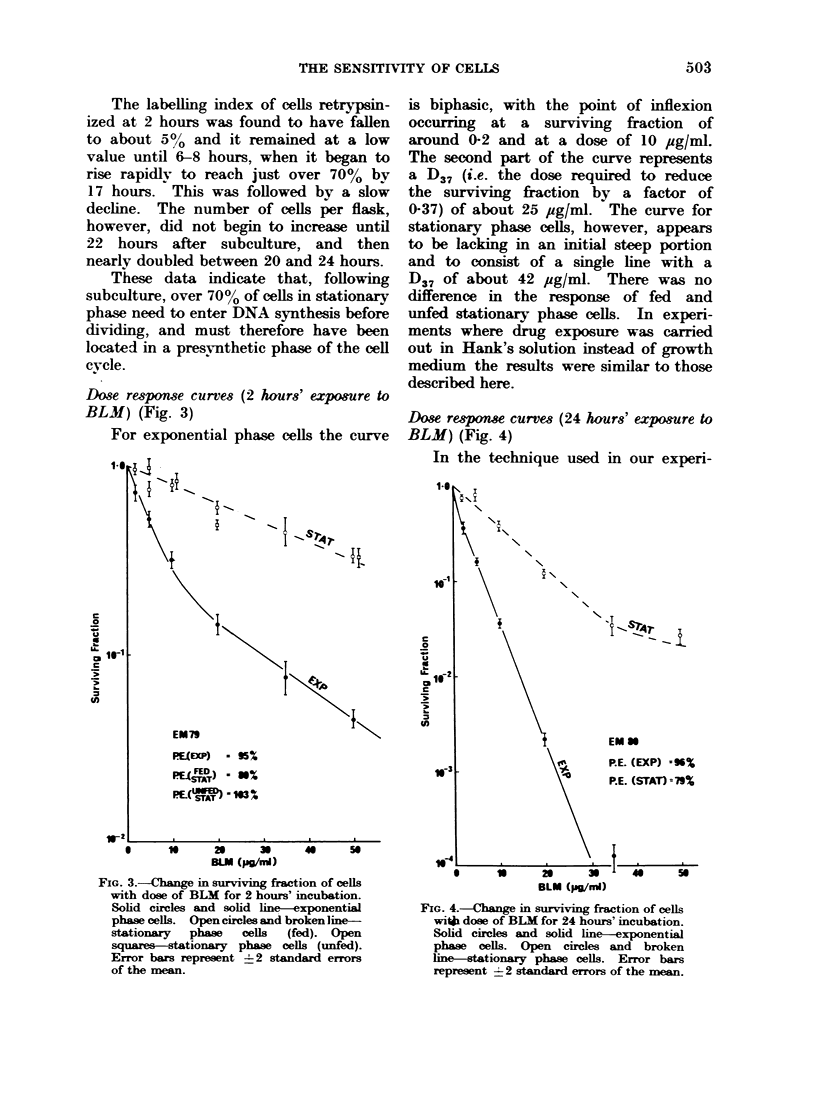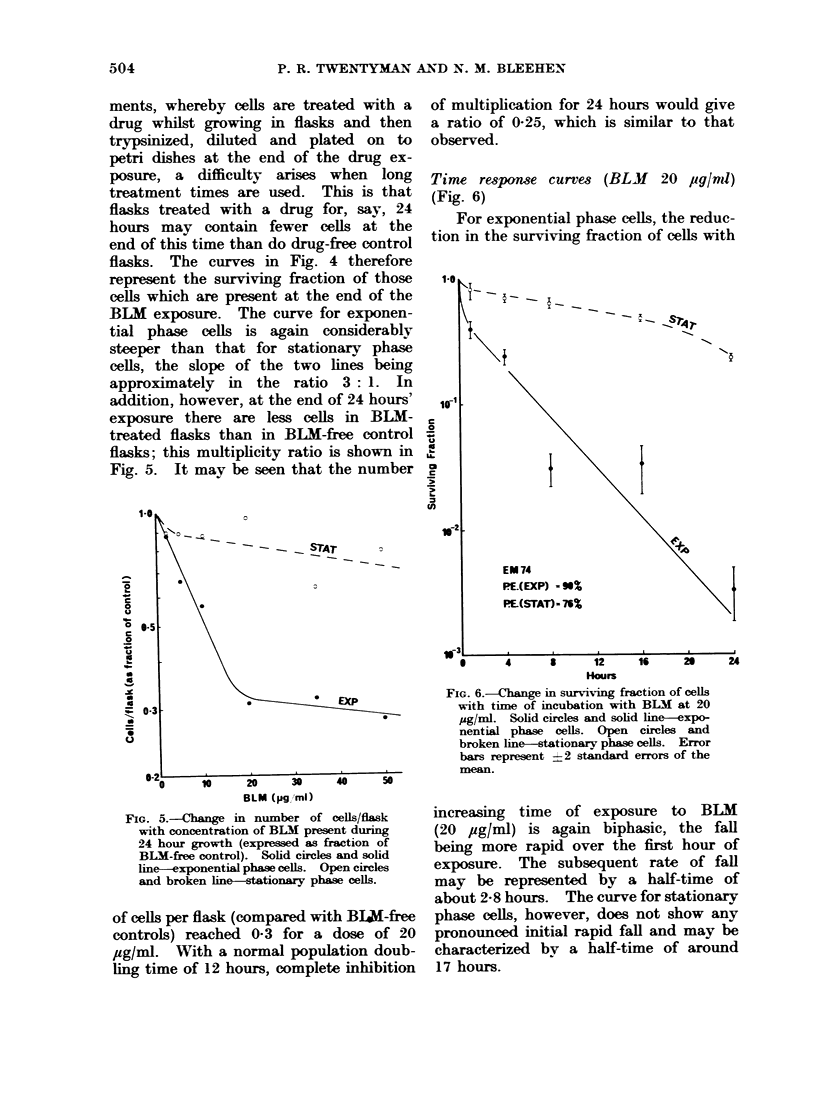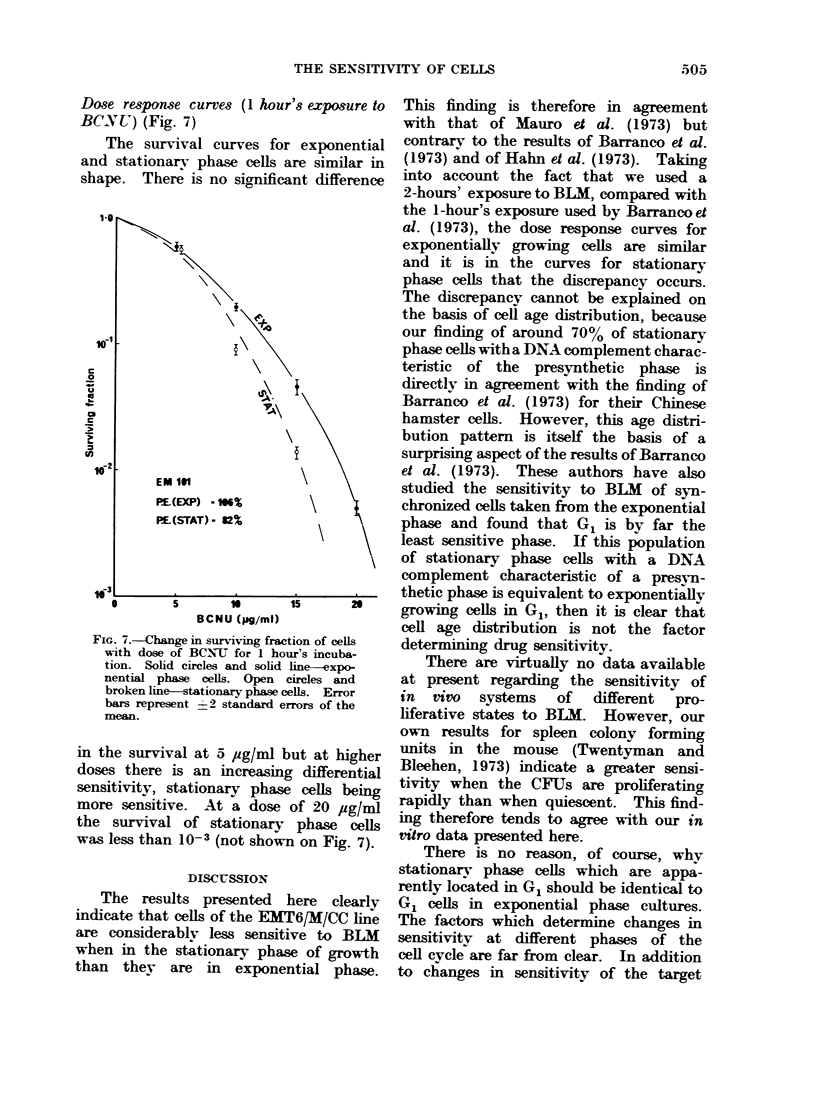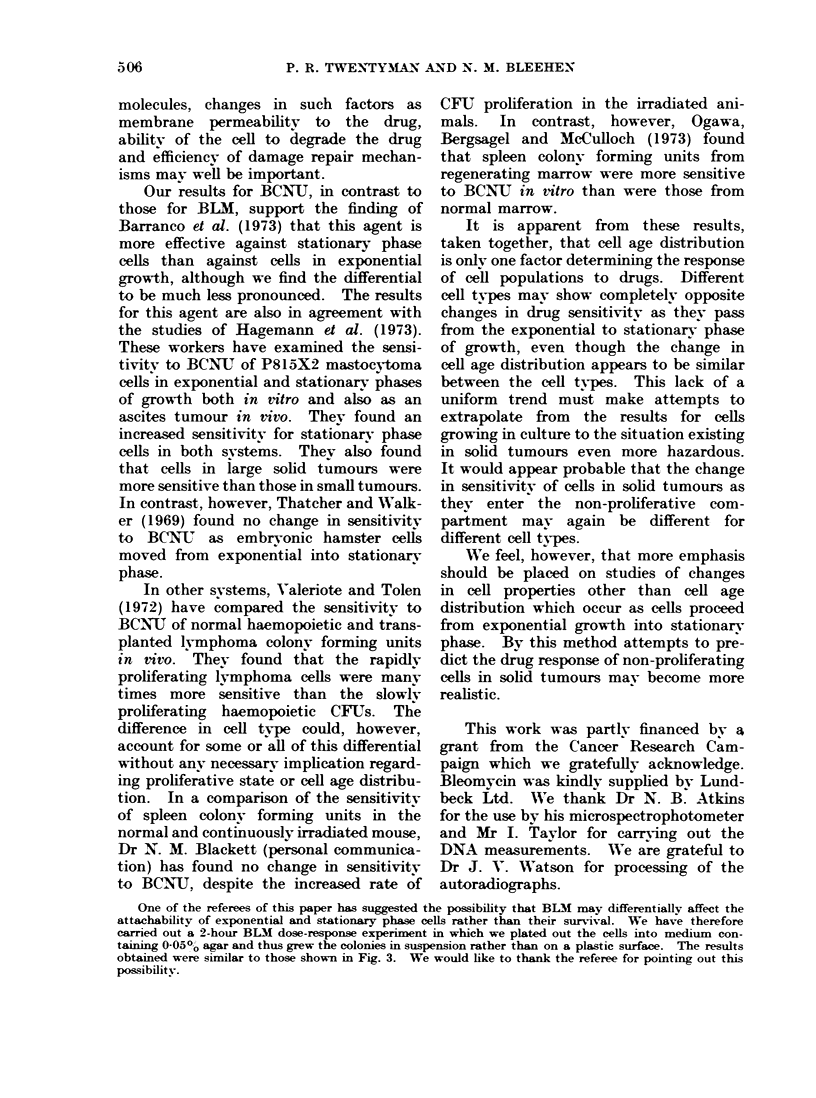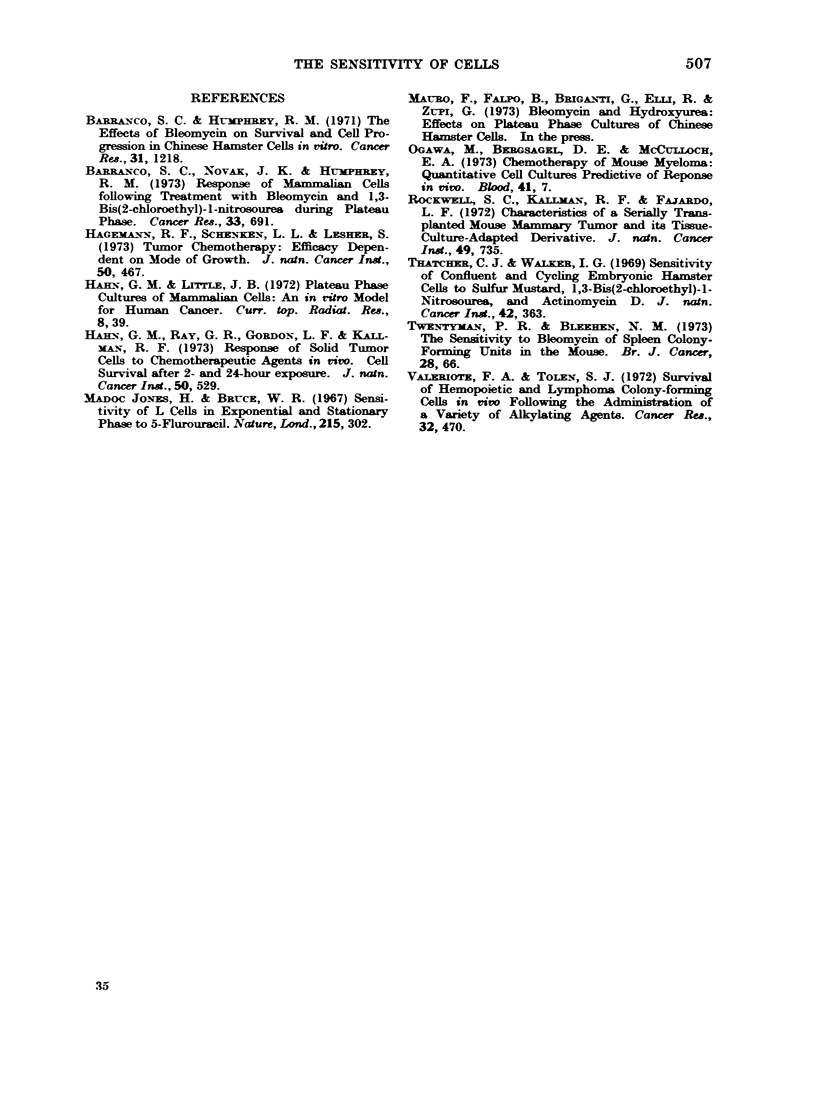# The Sensitivity of Cells in Exponential and Stationary Phases of Growth to Bleomycin and to 1,3-bis(2-chloroethyl)-1-Nitrosourea

**DOI:** 10.1038/bjc.1973.179

**Published:** 1973-12

**Authors:** P. R. Twentyman, N. M. Bleehen

## Abstract

Studies of EMT6 mouse tumour cells growing *in vitro* have shown that these cells become less sensitive to bleomycin as they pass from exponential growth into stationary phase. This result is the opposite of that recently reported by two other groups of workers using different cell systems, but in agreement with the results of a third group of workers. Results for 1,3-bis(2-chloroethyl)-1-nitrosourea, however, confirm the findings of other workers that cells become more sensitive to this agent as they pass into stationary phase.

These findings are discussed with particular reference to discrepancies which appear to exist between various cell systems.


*Br. J. Cancer* (1973) **28**, 500

### THE SENSITIVITY OF CELLS IN EXPONENTIAL AND STATIONARY PHASES OF GROWTH TO BLEOMYCIN AND TO 1,3-BIS(2-CHLOROETHYL)-1-NITROSOUREA

P. R. TWENTYMAN AND N. M. BLEEHEN

*From the Academic Department of Radiotherapy, The Middlesex Hospital Medical School, London W.1*

Received 9 July 1973. Accepted 31 August 1973

**Summary.—Studies of EMT6 mouse tumour cells growing *in vitro* have shown that these cells become less sensitive to bleomycin as they pass from exponential growth into stationary phase. This result is the opposite of that recently reported by two other groups of workers using different cell systems, but in agreement with the results of a third group of workers. Results for 1,3-bis(2-chloroethyl)-1-nitrosourea, however, confirm the findings of other workers that cells become more sensitive to this agent as they pass into stationary phase.**

**These findings are discussed with particular reference to discrepancies which appear to exist between various cell systems.**

THERE has been much interest recently in the radiation and drug response of cultured cells in the stationary (or plateau) phase of growth. Several comparisons have now been made of the sensitivity of cells in exponential and stationary phases of growth to chemotherapeutic agents (Madoc-Jones and Bruce, 1967; Thatcher and Walker, 1969; Hagemann, Schenken and Lesher, 1973).

We recently concluded a study of the response of EMT6 mouse tumour cells to bleomycin (BLM) when in these two phases of growth. Our results suggested that cells in stationary phase are much less sensitive to this agent than are exponentially growing cells. Subsequently, however, two groups of workers (Hahn *et al.*, 1973; Barranco, Novak and Humphrey, 1973) have reported the opposite findings in their studies using Chinese hamster cell lines. An observation similar to ours has, however, been made by Mauro *et al.* (1973) using V79-735B-(SS1) Chinese hamster cells.

In view of these contradictory observations, our data have been extended to include information regarding the age distribution of the EMT6/M/CC cells when in stationary phase. We have also studied the response of these cells to 1,3-bis(2-chloroethyl)-1-nitrosurea (BCNU), as this agent was also found by Barranco *et al.* (1973) to have considerably more effect on stationary cells in their system. It is clearly of interest to determine whether the discrepancy found for BLM also applies to BCNU.

### MATERIALS AND METHODS

*The cell line.*—The cells used in this study were designated EMT6/M/CC. These originated in a mouse alveolar tumour nodule; they were successively transplanted between animal and *in vitro* culture (Rockwell, Kallman and Fajardo, 1972) and then grown in continuous culture for about one year in this laboratory. Cells were cultured in 30 ml plastic tissue culture flasks (Falcon Plastics) containing 5 ml of Eagle's minimum essential medium supplemented with 20% calf serum.

Flasks were inoculated with either $10^5$ or $1{\cdot}5 \times 10^6$ cells and counts carried out at daily intervals afterwards, in order to determine the rate of increase in cell numbers. The cells were removed from the surface of the flasks using 0·05% trypsin for 15 min and counts were made using a haemacytometer. In some of the flasks the medium was changed daily from Day 2 (flasks inoculated at $1{\cdot}5 \times 10^6$ cells) or from Day 3 (flasks inoculated at $10^5$ cells).

*Cell-age distribution in stationary phase.*—The distribution of DNA content in exponential and stationary phase cells was defined using Feulgen staining and microspectrophotometry, and determinations were carried out on coverslip smears made immediately following trypsinization and resuspension of cells.

In addition, observations were made of the number of cells synthesizing DNA and the total number of cells recoverable from the flasks following resuspension of stationary phase cultures. Flasks were inoculated at about $5 \times 10^5$ cells taken immediately after trypsinization. At 2-hourly intervals, [$^3$H]TdR (2 $\mu$Ci/ml) was added to a flask and after 10 min the medium was changed and the culture retrypsinized. A count was carried out and then the slide preparations made using a cytocentrifuge (Shandon Ltd).

The slides were dipped in Ilford K5 nuclear emulsion, exposed for 3 days and developed in Kodak D19 developer. The proportion of labelled cells was determined by counting at least 200 cells/slide. The [$^3$H]TdR pulse labelling indices of exponential and stationary phase cells were also determined by the same technique.

*Drug response.*—Bleomycin (Batch F1921) was obtained as a freeze dried plug. This was dissolved in sterile water, kept in a deep freeze at −30°C and subsequently thawed and diluted in medium immediately before use. BCNU was kept at −70°C, dissolved in absolute ethanol at 5 mg/ml and diluted in medium before use. The appropriate dose of drug, in a volume of between 0·05 and 0·2 ml of medium, was added directly to the medium in which the cells were growing. In two BLM experiments, however, growth medium was removed from both exponential and stationary phase flasks and the drug was added in 5 ml of Hank's balanced salt solution.

After drug treatment cells were trypsinized from the flasks, counted, diluted and plated on to 50 mm tissue culture dishes (Sterilin Ltd). Dishes were kept for 11 days at 37°C and high humidity in plastic boxes gassed with a mixture of 95% air and 5% $CO_2$. At the end of this time the dishes were fixed in absolute alcohol, stained with crystal violet, and colonies containing more than 50 cells were counted.

### RESULTS

### *Multiplication of cells in culture*

The increase in numbers of cells in tissue culture flasks inoculated with $10^5$ cells from stationary phase cultures is shown in Fig. 1. For the first 24 hours there was a lag phase, during which time

FIG. 1.—Change in number of cells/flask with time after inoculation. Solid circles and solid line—flasks inoculated at $10^5$ cells without medium change. Open circles and dotted line—flasks inoculated at $10^5$ cells with daily medium change from Day 3. Squares and broken line—flasks inoculated at $1{\cdot}5 \times 10^6$ cells with daily medium change from Day 2.

no increase in numbers occurred. Over the subsequent 3 days the increase in numbers was exponential, with a cell doubling time of about 12 hours. In flasks where the medium was not changed a peak count of about $4 \times 10^6$ was reached on Day 4 and this was rapidly followed by degeneration and death of the whole population. When the medium was changed daily from Day 3, however, the number of cells in the flask reached about $9 \times 10^6$ and was maintained at this level for as long as observation was continued. In order to avoid the long delay in establishing stationary phase cultures, some flasks were seeded at $1{\cdot}5 \times 10^6$ cells and the medium changed on Day 2 and each subsequent day. These flasks reached the same plateau level as flasks seeded at lower cell numbers.

In all the experiments to be described, therefore, exponential phase cultures were inoculated at least 48 hours previously and contained between $3 \times 10^5$ and $10^6$ cells/flask at the time of the experiment. One experiment with BLM was carried out on unfed stationary phase cells at about $4 \times 10^6$ cells/flask. In the remainder of the experiments, stationary phase cultures were inoculated at $1{\cdot}5 \times 10^6$ cells/flask at least 4 days before the experiment and had been fed at daily intervals from Day 2, resulting in a cell yield of about $9 \times 10^6$ cells/flask.

*Microspectrophotometric measurements*

The results of these determinations indicated that in stationary phase cultures approximately 70% of cells have a $G_1$ complement of DNA.

*Labelling indices*

The [$^3$H]TdR pulse labelling indices for 5 exponential and 5 stationary (fed) phase flasks were determined and the mean values were found to be 52·2 ($\pm$1·3)% for exponential phase cultures and 26·4 ($\pm$1·4)% for stationary phase cultures (95% confidence limits in brackets). It is clear therefore that considerable proliferation is occurring in stationary phase cultures and that maintenance of constant cell number depends upon an equivalent amount of cell loss.

*Cell progression* (Fig. 2)

When $5 \times 10^5$ cells obtained by trypsinization of stationary phase cultures were inoculated into flasks and then retrypsinized between 4 and 20 hours later only about 50% of the cells were recovered. The reason for this is not clear although it is known that some cells are lost during the trypsinization procedure. A further factor may have been that the cells used in this experiment had been in stationary phase for about 5 days. For other cells it is known that the plating efficiency decreases as the cells remain in stationary phase for longer times (Hahn and Little, 1972).

FIG. 2.—Change in [$^3$H]TdR labelling index and in number of cells/flask with time after subculture. Solid circles and broken line—cells/flask. Open circles and solid line—labelling index.

The labelling index of cells retrypsinized at 2 hours was found to have fallen to about 5% and it remained at a low value until 6–8 hours, when it began to rise rapidly to reach just over 70% by 17 hours. This was followed by a slow decline. The number of cells per flask, however, did not begin to increase until 22 hours after subculture, and then nearly doubled between 20 and 24 hours.

These data indicate that, following subculture, over 70% of cells in stationary phase need to enter DNA synthesis before dividing, and must therefore have been located in a presynthetic phase of the cell cycle.

*Dose response curves* (*2 hours' exposure to BLM*) (Fig. 3)

For exponential phase cells the curve is biphasic, with the point of inflexion occurring at a surviving fraction of around 0·2 and at a dose of 10 μg/ml. The second part of the curve represents a $D_{37}$ (*i.e.* the dose required to reduce the surviving fraction by a factor of 0·37) of about 25 μg/ml. The curve for stationary phase cells, however, appears to be lacking in an initial steep portion and to consist of a single line with a $D_{37}$ of about 42 μg/ml. There was no difference in the response of fed and unfed stationary phase cells. In experiments where drug exposure was carried out in Hank's solution instead of growth medium the results were similar to those described here.

*Dose response curves* (*24 hours' exposure to BLM*) (Fig. 4)

In the technique used in our experi-

FIG. 3.—Change in surviving fraction of cells with dose of BLM for 2 hours' incubation. Solid circles and solid line—exponential phase cells. Open circles and broken line—stationary phase cells (fed). Open squares—stationary phase cells (unfed). Error bars represent ±2 standard errors of the mean.

FIG. 4.—Change in surviving fraction of cells with dose of BLM for 24 hours' incubation. Solid circles and solid line—exponential phase cells. Open circles and broken line—stationary phase cells. Error bars represent ±2 standard errors of the mean.

ments, whereby cells are treated with a drug whilst growing in flasks and then trypsinized, diluted and plated on to petri dishes at the end of the drug exposure, a difficulty arises when long treatment times are used. This is that flasks treated with a drug for, say, 24 hours may contain fewer cells at the end of this time than do drug-free control flasks. The curves in Fig. 4 therefore represent the surviving fraction of those cells which are present at the end of the BLM exposure. The curve for exponential phase cells is again considerably steeper than that for stationary phase cells, the slope of the two lines being approximately in the ratio 3 : 1. In addition, however, at the end of 24 hours' exposure there are less cells in BLM-treated flasks than in BLM-free control flasks; this multiplicity ratio is shown in Fig. 5. It may be seen that the number

FIG. 5.—Change in number of cells/flask with concentration of BLM present during 24 hour growth (expressed as fraction of BLM-free control). Solid circles and solid line—exponential phase cells. Open circles and broken line—stationary phase cells.

of cells per flask (compared with BLM-free controls) reached 0·3 for a dose of 20 μg/ml. With a normal population doubling time of 12 hours, complete inhibition of multiplication for 24 hours would give a ratio of 0·25, which is similar to that observed.

*Time response curves* (*BLM* 20 *μg/ml*) (Fig. 6)

For exponential phase cells, the reduction in the surviving fraction of cells with

FIG. 6.—Change in surviving fraction of cells with time of incubation with BLM at 20 μg/ml. Solid circles and solid line—exponential phase cells. Open circles and broken line—stationary phase cells. Error bars represent ±2 standard errors of the mean.

increasing time of exposure to BLM (20 μg/ml) is again biphasic, the fall being more rapid over the first hour of exposure. The subsequent rate of fall may be represented by a half-time of about 2·8 hours. The curve for stationary phase cells, however, does not show any pronounced initial rapid fall and may be characterized by a half-time of around 17 hours.

*Dose response curves* (1 *hour's exposure to BCNU*) (Fig. 7)

The survival curves for exponential and stationary phase cells are similar in shape. There is no significant difference

FIG. 7.—Change in surviving fraction of cells with dose of BCNU for 1 hour's incubation. Solid circles and solid line—exponential phase cells. Open circles and broken line—stationary phase cells. Error bars represent ±2 standard errors of the mean.

in the survival at 5 μg/ml but at higher doses there is an increasing differential sensitivity, stationary phase cells being more sensitive. At a dose of 20 μg/ml the survival of stationary phase cells was less than $10^{-3}$ (not shown on Fig. 7).

### DISCUSSION

The results presented here clearly indicate that cells of the EMT6/M/CC line are considerably less sensitive to BLM when in the stationary phase of growth than they are in exponential phase. This finding is therefore in agreement with that of Mauro *et al.* (1973) but contrary to the results of Barranco *et al.* (1973) and of Hahn *et al.* (1973). Taking into account the fact that we used a 2-hours' exposure to BLM, compared with the 1-hour's exposure used by Barranco *et al.* (1973), the dose response curves for exponentially growing cells are similar and it is in the curves for stationary phase cells that the discrepancy occurs. The discrepancy cannot be explained on the basis of cell age distribution, because our finding of around 70% of stationary phase cells with a DNA complement characteristic of the presynthetic phase is directly in agreement with the finding of Barranco *et al.* (1973) for their Chinese hamster cells. However, this age distribution pattern is itself the basis of a surprising aspect of the results of Barranco *et al.* (1973). These authors have also studied the sensitivity to BLM of synchronized cells taken from the exponential phase and found that $G_1$ is by far the least sensitive phase. If this population of stationary phase cells with a DNA complement characteristic of a presynthetic phase is equivalent to exponentially growing cells in $G_1$, then it is clear that cell age distribution is not the factor determining drug sensitivity.

There are virtually no data available at present regarding the sensitivity of *in vivo* systems of different proliferative states to BLM. However, our own results for spleen colony forming units in the mouse (Twentyman and Bleehen, 1973) indicate a greater sensitivity when the CFUs are proliferating rapidly than when quiescent. This finding therefore tends to agree with our *in vitro* data presented here.

There is no reason, of course, why stationary phase cells which are apparently located in $G_1$ should be identical to $G_1$ cells in exponential phase cultures. The factors which determine changes in sensitivity at different phases of the cell cycle are far from clear. In addition to changes in sensitivity of the target molecules, changes in such factors as membrane permeability to the drug, ability of the cell to degrade the drug and efficiency of damage repair mechanisms may well be important.

Our results for BCNU, in contrast to those for BLM, support the finding of Barranco *et al.* (1973) that this agent is more effective against stationary phase cells than against cells in exponential growth, although we find the differential to be much less pronounced. The results for this agent are also in agreement with the studies of Hagemann *et al.* (1973). These workers have examined the sensitivity to BCNU of P815X2 mastocytoma cells in exponential and stationary phases of growth both *in vitro* and also as an ascites tumour *in vivo*. They found an increased sensitivity for stationary phase cells in both systems. They also found that cells in large solid tumours were more sensitive than those in small tumours. In contrast, however, Thatcher and Walker (1969) found no change in sensitivity to BCNU as embryonic hamster cells moved from exponential into stationary phase.

In other systems, Valeriote and Tolen (1972) have compared the sensitivity to BCNU of normal haemopoietic and transplanted lymphoma colony forming units *in vivo*. They found that the rapidly proliferating lymphoma cells were many times more sensitive than the slowly proliferating haemopoietic CFUs. The difference in cell type could, however, account for some or all of this differential without any necessary implication regarding proliferative state or cell age distribution. In a comparison of the sensitivity of spleen colony forming units in the normal and continuously irradiated mouse, Dr N. M. Blackett (personal communication) has found no change in sensitivity to BCNU, despite the increased rate of CFU proliferation in the irradiated animals. In contrast, however, Ogawa, Bergsagel and McCulloch (1973) found that spleen colony forming units from regenerating marrow were more sensitive to BCNU *in vitro* than were those from normal marrow.

It is apparent from these results, taken together, that cell age distribution is only one factor determining the response of cell populations to drugs. Different cell types may show completely opposite changes in drug sensitivity as they pass from the exponential to stationary phase of growth, even though the change in cell age distribution appears to be similar between the cell types. This lack of a uniform trend must make attempts to extrapolate from the results for cells growing in culture to the situation existing in solid tumours even more hazardous. It would appear probable that the change in sensitivity of cells in solid tumours as they enter the non-proliferative compartment may again be different for different cell types.

We feel, however, that more emphasis should be placed on studies of changes in cell properties other than cell age distribution which occur as cells proceed from exponential growth into stationary phase. By this method attempts to predict the drug response of non-proliferating cells in solid tumours may become more realistic.

This work was partly financed by a grant from the Cancer Research Campaign which we gratefully acknowledge. Bleomycin was kindly supplied by Lundbeck Ltd. We thank Dr N. B. Atkins for the use by his microspectrophotometer and Mr I. Taylor for carrying out the DNA measurements. We are grateful to Dr J. V. Watson for processing of the autoradiographs.

One of the referees of this paper has suggested the possibility that BLM may differentially affect the attachability of exponential and stationary phase cells rather than their survival. We have therefore carried out a 2-hour BLM dose-response experiment in which we plated out the cells into medium containing 0·05% agar and thus grew the colonies in suspension rather than on a plastic surface. The results obtained were similar to those shown in Fig. 3. We would like to thank the referee for pointing out this possibility.

### REFERENCES

BARRANCO, S. C. & HUMPHREY, R. M. (1971) The Effects of Bleomycin on Survival and Cell Progression in Chinese Hamster Cells *in vitro*. *Cancer Res.*, **31**, 1218.

BARRANCO, S. C., NOVAK, J. K. & HUMPHREY, R. M. (1973) Response of Mammalian Cells following Treatment with Bleomycin and 1,3-Bis(2-chloroethyl)-1-nitrosourea during Plateau Phase. *Cancer Res.*, **33**, 691.

HAGEMANN, R. F., SCHENKEN, L. L. & LESHER, S. (1973) Tumor Chemotherapy: Efficacy Dependent on Mode of Growth. *J. natn. Cancer Inst.*, **50**, 467.

HAHN, G. M. & LITTLE, J. B. (1972) Plateau Phase Cultures of Mammalian Cells: An *in vitro* Model for Human Cancer. *Curr. top. Radiat. Res.*, **8**, 39.

HAHN, G. M., RAY, G. R., GORDON, L. F. & KALLMAN, R. F. (1973) Response of Solid Tumor Cells to Chemotherapeutic Agents *in vivo*. Cell Survival after 2- and 24-hour exposure. *J. natn. Cancer Inst.*, **50**, 529.

MADOC JONES, H. & BRUCE, W. R. (1967) Sensitivity of L Cells in Exponential and Stationary Phase to 5-Flurouracil. *Nature, Lond.*, **215**, 302.

MAURO, F., FALPO, B., BRIGANTI, G., ELLI, R. & ZUPI, G. (1973) Bleomycin and Hydroxyurea: Effects on Plateau Phase Cultures of Chinese Hamster Cells. In the press.

OGAWA, M., BERGSAGEL, D. E. & MCCULLOCH, E. A. (1973) Chemotherapy of Mouse Myeloma: Quantitative Cell Cultures Predictive of Reponse *in vivo*. *Blood*, **41**, 7.

ROCKWELL, S. C., KALLMAN, R. F. & FAJARDO, L. F. (1972) Characteristics of a Serially Transplanted Mouse Mammary Tumor and its Tissue-Culture-Adapted Derivative. *J. natn. Cancer Inst.*, **49**, 735.

THATCHER, C. J. & WALKER, I. G. (1969) Sensitivity of Confluent and Cycling Embryonic Hamster Cells to Sulfur Mustard, 1,3-Bis(2-chloroethyl)-1-Nitrosourea, and Actinomycin D. *J. natn. Cancer Inst.*, **42**, 363.

TWENTYMAN, P. R. & BLEEHEN, N. M. (1973) The Sensitivity to Bleomycin of Spleen Colony-Forming Units in the Mouse. *Br. J. Cancer*, **28**, 66.

VALERIOTE, F. A. & TOLEN, S. J. (1972) Survival of Hemopoietic and Lymphoma Colony-forming Cells *in vivo* Following the Administration of a Variety of Alkylating Agents. *Cancer Res.*, **32**, 470.